# Milrinone-Induced Pharmacological Preconditioning in Cardioprotection: Hints for a Role of Mitochondrial Mechanisms

**DOI:** 10.3390/jcm8040507

**Published:** 2019-04-12

**Authors:** Annika Raupach, Julia Reinle, Martin Stroethoff, Alexander Mathes, André Heinen, Markus W. Hollmann, Ragnar Huhn, Sebastian Bunte

**Affiliations:** 1 Department of Anesthesiology, University Hospital Duesseldorf, Moorenstr. 5, 40225 Duesseldorf, Germany; Annika.Raupach@med.uni-duesseldorf.de (A.R.); Julia.Reinle@uni-duesseldorf.de (J.R.); Ragnar.Huhn@med.uni-duesseldorf.de (R.H.); Sebastian.Bunte@med.uni-duesseldorf.de (S.B.); 2 Department of Anesthesiology and Intensive Care Medicine, University Hospital Cologne, Kerpener Str. 62, 50937 Cologne, Germany; Alexander.Mathes@uk-koeln.de; 3 Institute of Cardiovascular Physiology, Heinrich-Heine-University Duesseldorf, Universitaetsstr. 1, 40225 Duesseldorf, Germany; Andre.Heinen@uni-duesseldorf.de; 4 Department of Anesthesiology, Amsterdam University Medical Center (AUMC), Location AMC, Meiberdreef 9, 1105 AZ Amsterdam, The Netherlands; M.W.Hollmann@amc.uva.nl

**Keywords:** milrinone, preconditioning, cardioprotection, reperfusion injury, mPTP

## Abstract

The activation of mitochondrial calcium-sensitive potassium (mBK_Ca_) channels is crucially involved in cardioprotection induced by preconditioning. For milrinone (Mil)-induced preconditioning, the involvement of mBK_Ca_-channels and further mitochondrial signaling is unknown. We hypothesize that (1) Mil-induced preconditioning is concentration-dependent and (2) that the activation of mBK_Ca_-channels, release of reactive oxygen species (ROS), and the mitochondrial permeability transition pore (mPTP) could be involved. Isolated hearts of male Wistar rats were perfused with Krebs-Henseleit buffer and underwent 33 min of ischemia followed by 60 min of reperfusion. For determination of a concentration-dependent effect of Mil, hearts were perfused with different concentrations of Mil (0.3–10 µM) over 10 min before ischemia. In a second set of experiments, in addition to controls, hearts were pretreated with the lowest protective concentration of 1 µM Mil either alone or combined with the mBK_Ca_-channel blocker paxilline (Pax + Mil), or paxilline alone (Pax). In additional groups, Mil was administered with and without the ROS scavenger N-2-mercaptopropionylglycine (MPG + Mil, MPG) or the mPTP inhibitor cyclosporine A (MPG + Mil + CsA, CsA + Mil), respectively. Infarct sizes were determined by triphenyltetrazolium chloride (TTC) staining. The lowest and most cardioprotective concentration was 1 µM Mil (Mil 1: 32 ± 6%; *p* < 0.05 vs. Con: 63 ± 8% and Mil 0.3: 49 ± 6%). Pax and MPG blocked the infarct size reduction of Mil (Pax + Mil: 53 ± 6%, MPG + Mil: 59 ± 7%; *p* < 0.05 vs. Mil: 34 ± 6%) without having an effect on infarct size when administered alone (Pax: 53 ± 7%, MPG: 58 ± 5%; ns vs. Con). The combined administration of CsA completely restored the MPG-inhibited cardioprotection of Mil (MPG + Mil + CsA: 35 ± 7%, *p* < 0.05 vs. MPG + Mil). Milrinone concentration-dependently induces preconditioning. Cardioprotection is mediated by the activation of mBK_Ca_-channels, release of ROS and mPTP inhibition.

## 1. Introduction

Milrinone (Mil) is a phosphodiesterase 3 (PDE3) inhibitor which is used in cardiac surgeries as an inotropic and pulmonary vasodilator agent [1]. Inhibition of PDE3 increases the concentration of cyclic adenosine monophosphate (cAMP) [2], resulting in the activation of protein kinase A (PKA), followed by an increase of intracellular calcium (Ca^2+^) levels [3]. In 2001, Sanada et al. firstly described a cardioprotective effect of Mil preconditioning on ischemia/reperfusion (I/R) injury in a canine model [3]. The cardioprotection by Mil was mediated by a cAMP-, PKA-, and mitogen-activated protein kinase (MAPK)-dependent mechanism. A protective effect of Mil preconditioning on I/R injury was also shown for liver and brain. Kume et al. improved hepatic microcirculation after warm I/R injury in a rat model via pretreatment with Mil [4]. A neuroprotective effect of Mil by pharmacological preconditioning was shown in a mouse model of 24 h I/R cerebral injury [5]. Mil reduced cerebral infarct sizes and reversed I/R-induced impairments of memory and motor coordination.

Recently, we showed that postconditioning with Mil induces cardioprotection, requiring the activation of mitochondrial large conductance calcium-sensitive potassium (mBK_Ca_) channels in an in-vitro rat model [6], indicating a crucial role for mitochondria in Mil postconditioning. For myocardial conditioning, it is suggested that mitochondria serve as the common target of cardioprotective signaling, whereas different signaling pathways into and inside mitochondria influence mitochondrial permeability transition pore (mPTP) opening (reviewed in [7]). mPTP opening at the onset of reperfusion after ischemia is crucial for cell death in myocardial injury [8]. Inhibition of mPTP opening by ischemic as well as pharmacological-induced conditioning results in reduced infarct sizes [9]. Matsumoto et al. demonstrated that Mil postconditioning is also mediated by inhibition of mPTP opening [10]. Amongst others, radical oxygen species (ROS) induce mPTP opening [7]. During ischemia, ROS formation is mostly induced by mitochondria and is further increased at the onset of reperfusion [7]. High amounts of ROS are detrimental for I/R injury. However, low amounts of ROS during ischemic preconditioning (IPC) are essential for the IPC-induced infarct size reduction [11].

Although Sanada et al. described the first steps in the signaling pathway of Mil preconditioning, the following signaling into and inside mitochondria was not the objective of their study. Therefore, we set out to determine the lowest cardioprotective concentration of Mil and the underlying mechanisms involved in Mil-induced preconditioning. We hypothesize that cardioprotection by Mil is concentration-dependent and that the activation of mBK_Ca_-channels, ROS release and the mPTP is involved in this phenomenon.

## 2. Material and Methods

The study was conducted on the baseline of the Guide for the Care and Use of Laboratory Animals published by the National Institutes of Health (Publication number 85-23, revised 1996). The animal experiments were approved by the Animal Ethics Committee of the University of Duesseldorf, Germany (project number O27/12).

### 2.1. Surgical Preparation

The procedure was performed as described in detail previously [12]. Male Wistar rats (2–3 months old) were randomized into the respective groups and anesthetized with an intraperitoneal injection of pentobarbital (80 mg/kg body weight; Narcoren, Merial, Germany). After decapitation by a guillotine and thoracotomy, hearts were isolated and mounted on a Langendorff system with constant pressure (80 mmHg) and perfused with a modified Krebs-Henseleit solution (KHB: 116 mM NaCl, 4.7 mM KCl, 1.1 mM MgSO_4_, 1.17 mM KH_2_PO_4_, 24.9 mM NaHCO_3_, 2.5 mM CaCl_2_, 8.3 mM glucose, and 2.2 mM pyruvate at 37 °C). Pressure measurements were realized via a fluid-filled balloon. The end-diastolic pressure was set at 1–4 mmHg. All hearts underwent the following protocol: 20 min of equilibration, 10 min application of substances, 33 min of ischemia, 60 min of reperfusion. Hemodynamic variables were measured continuously, digitized using an analogue-to-digital converter (PowerLab/8SP, ADInstruments Pty Ltd, Castle Hill, Australia) at a sampling rate of 500 Hz, and recorded on a personal computer using Chart for Windows v5.0 (ADInstruments Pty Ltd, Castle Hill, Australia).

After reperfusion, hearts were stained with 0.75% triphenyltetrazoliumchloride (TTC) solution and the infarct size measurement was determined by a blinded investigator using planimetry [13].

### 2.2. Experimental Protocol

All hearts in both parts of the study underwent 33 min of global ischemia and 60 min of reperfusion.

**Figure 1:**
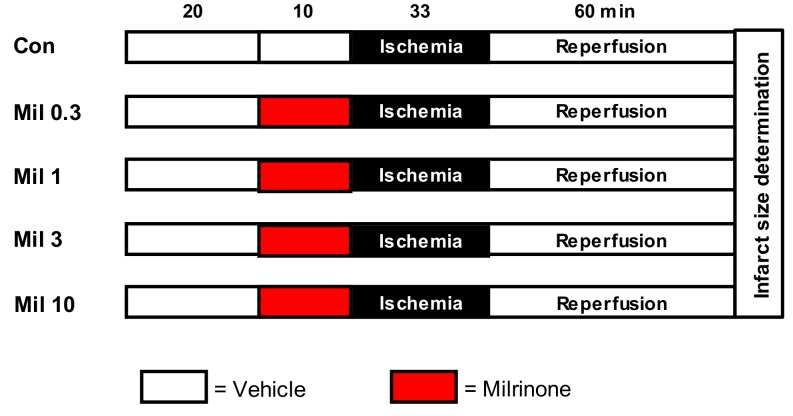
Experimental scheme from part one of the study: Con = Control; Mil = Milrinone.

In the first part of this study, we determined a concentration-dependent effect of milrinone. We randomly assigned the hearts to five groups (*n* = 7–9 per group, Figure 1):

- Control (Con): Hearts received no further treatment.
- Milrinone (Mil): Hearts were perfused with 0.3, 1, 3, and 10 µM Mil for 10 min before ischemia.

**Figure 2:**
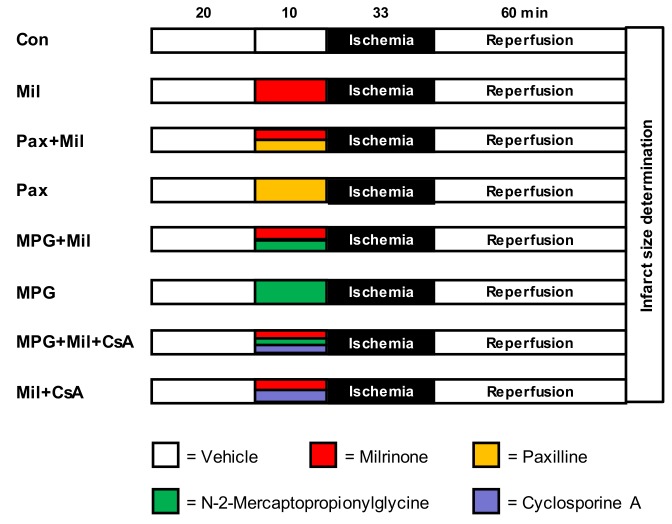
Experimental scheme from part two of the study: Con = Control; Mil = Milrinone; Pax = Paxilline; MPG = N-2-mercaptopropionylglycine; CsA = Cyclosporine A.

The second part of the study was designed to investigate the underlying mechanism of Mil-induced preconditioning. Based on the results of part one, we used a concentration of 1 µM Mil as that was the lowest cardioprotective concentration. Rat hearts were randomly assigned to eight groups (*n* = 7–9 per group, Figure 2):

- Control (Con): Hearts received no further treatment.
- Milrinone (Mil): Hearts were perfused with 1 µM Mil only for 10 min before ischemia.
- Paxilline + milrinone (Pax + Mil): Hearts were perfused with the mBK_Ca_-channel inhibitor Pax (1 µM) [6] combined with 1 µM Mil for 10 min before ischemia.
- Paxilline (Pax): Hearts were perfused with 1 µM Pax only for 10 min.
- N-2-mercaptopropionylglycine + milrinone (MPG + Mil): Hearts were perfused with the ROS sc#avenger MPG (1 mM) [14] combined with 1 µM Mil for 10 min before ischemia.
- N-2-mercaptopropionylglycine (MPG): Hearts were perfused with 1 mM MPG only for 10 min.
- N-2-mercaptopropionylglycine + milrinone + cyclosporine A (MPG + Mil + CsA): Hearts were perfused with 1 mM MPG [14] combined with 1 µM Mil and 0.2 µM CsA [15] for 10 min before ischemia.
- Milrinone + cyclosporine A (Mil + CsA): Hearts were perfused with 1 µM Mil and 0.2 µM CsA [15] for 10 min before ischemia.

After reperfusion, hearts were weighed to evaluate the wet weight and cut into 7 transverse slices (1–2 mm), starting from the cardiac apex to just before the cardiac valvular plane. The slices were stained for 15 min with 0.75% TTC solution and fixed in HCl for 24 h. The slices were scanned und the size of the infarcted area was determined by planimetry using SigmaScan Pro 5 computer software (SPSS Science Software, Chicago, IL, USA). At the end, heart slices were dried and weighted for dry weights.

### 2.3. Statistical Analysis

Sample size calculations revealed a group size of *n* = 8 for detecting a 25% mean difference and a standard deviation of 13% in infarct size with a power of 80% (*α* < 0.05 (two-tailed)).

Data are presented as mean ± standard deviation (SD). To compare hemodynamic variables between groups or between different time points within groups, we used a two-way analysis of variance (ANOVA) and a Tukey post hoc test (GraphPad Software, San Diego, CA, USA). An investigator blinded to the experimental groups determined the infarct sizes. A one-way ANOVA was chosen, followed by a Tukey post hoc test to analyze infarct size. Changes were regarded statistically significant if *p* < 0.05.

## 3. Results

### 3.1. Animal Characteristics

Table 1 shows body weight, wet and dry heart weight and level and time of maximal ischemic contracture of both parts of the study.

### 3.2. Infarct Size—Concentration Effect of Milrinone Preconditioning

**Figure 3:**
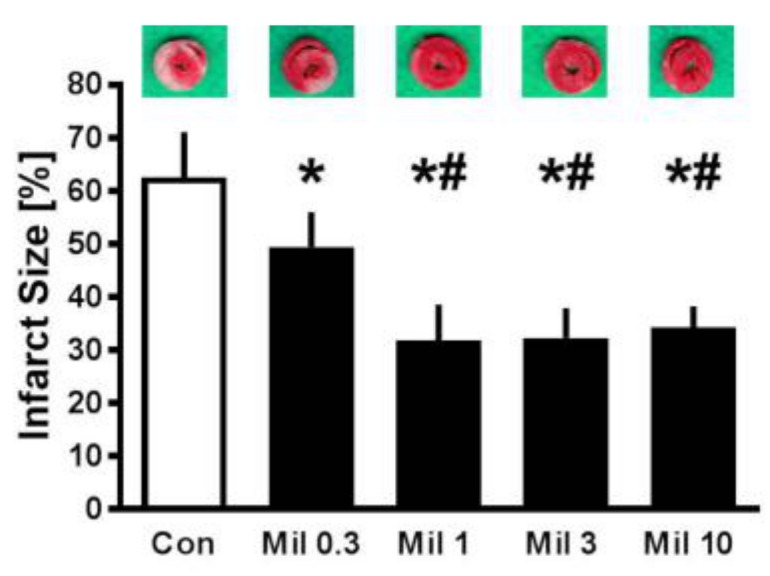
Infarct size measurement. The infarct size of controls (Con) and different concentrations of milrinone (Mil) preconditioning. For each group, an exemplary heart slice is shown. Data are mean ± SD. * *p* < 0.05 vs. Con, ^#^ *p* < 0.05 vs. Mil.

To determine the lowest and most cardioprotective dose of Mil preconditioning, rat hearts were preconditioned with different concentrations of Mil followed by infarct size determination. As shown in Figure 3, the infarct size in the control group was 63 ± 8% (Con). Preconditioning with 0.3 µM Mil reduced the infarct size significantly compared to the control (Mil 0.3: 49 ± 6%, *p* < 0.05 vs. Con). The 1 µM Mil concentration reduced the infarct size to a greater extent than 0.3 µM Mil (Mil 1: 32 ± 6%; *p* < 0.05 vs. Mil 0.3), while higher Mil concentrations were not able to further reduce the infarct size (Mil 3: 32 ± 6%, Mil 10: 34 ± 4%; ns vs. Mil 1). In further experiments, 1 µM Mil was used as the lowest and most cardioprotective dose of Mil.

### 3.3. Infarct Size—Mechanism of Milrinone Preconditioning

**Figure 4:**
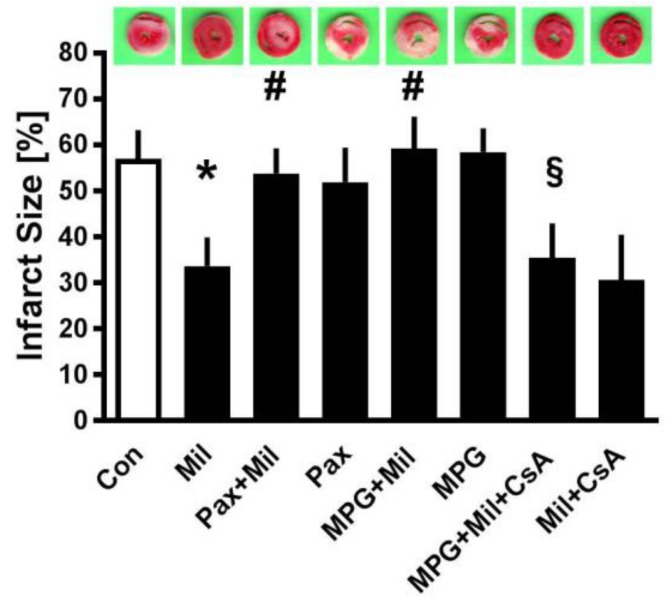
Infarct size measurement. The infarct size of controls (Con) and preconditioning with milrinone (Mil), with or without the mBK_Ca_-channel inhibitor paxilline (Pax), the ROS scavenger N-2-mercaptopropionylglycine (MPG) and/or the mPTP inhibitor Cyclosporine A (CsA). For each group, an exemplary heart slice is shown. Data are mean ± SD. * *p* < 0.05 vs. Con, ^#^ *p* < 0.05 vs. Mil, ^§^ *p* < 0.05 vs. MPG + Mil.

In the second part, the underlying mechanism of Mil preconditioning was determined. Mil reduced the reproducible infarct size in preconditioned hearts compared to that of Con hearts (Con: 57 ± 6% vs. Mil: 34 ± 6%; *p* < 0.05, Figure 4). Mil failed to reduce the infarct size both in combination with the mBK_Ca_-blocker Pax and with the radical scavenger MPG (Pax + Mil: 53 ± 6%, MPG + Mil: 59 ± 7%; ns. vs. Con). Furthermore, Pax and MPG did not have an effect on infarct sizes when administered alone (Pax: 53 ± 7%, MPG: 58 ± 5%; ns. vs. Con). The additional administration of the mPTP inhibitor CsA in the presence of Mil and MPG reduced the infarct size (MPG + Mil + CsA: 35 ± 7%; *p* < 0.05 vs. MPG + Mil), whereas CsA administration did not further enhance the infarct size reduction of Mil alone (Mil + CsA: 31 ± 10%; ns. vs. Mil). Recently, we demonstrated that CsA alone induced infarct size reduction in a comparable experimental setup in isolated rat heart [15]. Due to animal ethics reasons, we did not include this group.

### 3.4. Cardiac Function

Hemodynamic data from part one of the study are shown in Table 2 and data from part two are shown in Table 3. In part one of the study, phasic left ventricular pressure (LVP) and coronary flow were statistically different from baseline after ischemia and during reperfusion. Coronary flow was significantly different in the Mil 0.3 and Mil 10 group compared to Con before ischemia. The hemodynamic variables of part two of the study demonstrated a similar picture. Phasic LVP and coronary flow were statistically different from baseline after ischemia and during reperfusion. Before ischemia, phasic LVP was significantly different in the Pax + Mil and MPG + Mil + CsA groups compared to Con. No differences were determined in heart rate between the experimental groups in part two.

## 4. Discussion

The results of this study show that preconditioning with Mil has a concentration-dependent cardioprotective effect. The 1 µM Mil concentration represents the lowest and most cardioprotective concentration. The underlying mechanism of Mil preconditioning is mediated via the activation of mBK_Ca_-channels and the release of free oxygen radicals. Inhibition of mPTP opening seems to play a crucial role in the context of Mil-induced preconditioning.

Mil preconditioning revealed a concentration-dependent effect. The infarct size reduction by Mil 0.3 µM was amplified by 1 µM Mil, but higher Mil concentrations (3, 10 µM) did not induce a further infarct size reduction. Although it is unlikely, we cannot rule out that higher concentrations than 10 µM of Mil might induce a more pronounced infarct size-reducing effect. Previously, we demonstrated that Mil administration after global ischemia and at the onset of reperfusion was also cardioprotective [6]. Here, the lowest cardioprotective dose was 3 µM Mil, whereas 0.3 and 1 µM Mil was not protective. Thus, preconditioning needs lower concentrations of Mil to reduce infarct size than postconditioning with Mil.

The lowest and most cardioprotective concentration of 1 µM (211 ng/mL) Mil in the present study is at the lower limit of clinically relevant Mil plasma levels. In humans, plasma concentrations of 100–300 ng/mL (0.5–1.4 µM) Mil improve cardiac index in a dose-dependent manner, whereas concentrations above 500 ng/mL (2.4 µM) had only little additional benefit [16,17]. Interestingly, in patients with stage D heart failure, a plasma concentration of 450 ng/µL (2.1 µM) Mil was measured using a mean infusion rate of 0.4 µg/kg/min [17], which is a clinically used maintenance dosage in the upper range. An in vivo rat study showed an infarct size reduction using an infusion rate of 5 µg/kg/min Mil over 10 min (1/10 of the recommended loading dose) starting 5 min before reperfusion [18]. Thus, the required 1 µM Mil plasma concentration to induce cardioprotection will be easily reached by clinically used dosages.

In the present study, the mBK_Ca_-channel blocker Pax inhibited the infarct size-reducing effect of Mil, indicating that mitochondria are involved in the underlying mechanism via the activation of mBK_Ca_-channels. Pax is an indole diterpene belonging to a family of tremorgenic mycotoxins from *Penicillium paxilli* and serves as a positive allosteric modulator of BK_Ca-_channels [19]. Pax binds to the α-subunit of BK_Ca_-channels (K_i_ = 1.9 nM for block of currents in α-subunit-expressing oocytes) and enhances the binding of charybdotoxin to BK_Ca_-channels in vascular smooth muscle [20]. Inhibition by Pax involves a strong stabilization of closed conformations and Pax binds more tightly to the closed conformation, favoring occupancy of closed-channel conformations, and it is proposed that it binds to a superficial position near the entrance to the central cavity [21]. We used Pax in a concentration of 1 µM, which is sufficient for the specific inhibition of BK_Ca_-channels. Saleem et al. demonstrated that Pax inhibits splice variants of BK_Ca_-channels with similar IC_50_s (0.35–0.70 µmol·L^−1^) [22]. Pax also inhibits different isoforms of sarco/endoplasmic reticulum Ca^2+^-ATPase ( SERCA) with IC_50_s ranging between 5 and 50 µM [23], thus at least five times higher than that used in our experiments. Similar effects were reported for cerebellar inositol 1,4,5-trisphophate (InsP_3_) receptors. Pax, as a reversible inhibitor, inhibits the amount or extent of InsP_3_-induced Ca^2+^ release, at sub-maximal concentrations of InsP_3_, in a biphasic manner consistent with two inhibition constants (*K*_i_’s 6.7 and ≥400 μM) [24]. Taken together, a treatment with 1 µM Pax should result in specific inhibition of BK_Ca_-channels.

The role of mBK_Ca_-channels in cardioprotection is controversially discussed. Nevertheless, existing literature underline the importance of these channels in cardioprotection [25]. For example, IPC failed to reduce infarct size in *KCNMA1^-/-^* mice compared to wildtype mice [26]. *KCNMA1^-/-^* mice carry a global inactivation of the *KCNMA1* gene, which encodes the pore-forming α-subunit of BK_Ca_-channels [27]. A role of mBK_Ca_-channels in cardioprotection is also supported by studies employing the BK_Ca_-channel activator NS1619. The latter protects the heart from I/R injury in various species [25], for example in mice and rats [28,29]. The importance of mBK_Ca_-channels in cardioprotection is further supported by its preserved cardioprotective effect in the aged heart. Heinen et al. showed a reduction of infarct sizes by BK_Ca_-channel activator NS1619 in both young and aged rats [29], indicating an age-independent cardioprotection. In contrast, ischemic and pharmacological pre as well as postconditioning failed in the aged myocardium (reviewed in [30]).

The opening of mBK_Ca_-channels influences, amongst others, levels of ROS. A loss of mBK_Ca_-channels increases post-anoxic ROS levels in isolated cardiomyocyte mitochondria of *KCNMA1^-/-^* mice [26]. Heinen et al. demonstrated that NS1619 decreased ROS production in cardiac mitochondria, whereas Pax incompletely inhibited the reduction in ROS formation [31]. In the present study, the ROS scavenger MPG abolished the infarct size reduction by Mil, indicating a crucial role for ROS in cardioprotection induced by Mil preconditioning. Thus, ROS production by mBK_Ca_-channel activation is involved in Mil cardioprotection. This finding supports previous studies postulating that the opening of mBK_Ca_-channels influences ROS levels. Baines et al. [32] support our findings with the ROS scavenger MPG in an isolated, perfused rabbit heart model. Treatment with 300 µM MPG (infusion over 15 min, starting 20 min before regional ischemia) abolished infarct size reduction by ischemic preconditioning, whereas MPG had no effect on infarct size when administered alone. Data from an in vivo rat study revealed similar results with MPG [33]. Vigneron et al. demonstrated that 1 mM MPG inhibited infarct size reduction by ischemic preconditioning or pharmacological preconditioning by the selective mitochondrial adenosine triphosphate-dependent potassium (mitoK_ATP_) activator diazoxide in an isolated perfused mouse heart model [34]. Tanonaka et al. showed contradictory results to our study with an infarct size reduction by MPG alone [35]. The results of the study by Tanonaka et al. [35] are not comparable to our study due to small but important differences in the study design. In this study, the same concentration of 1 mM MPG was used, but MPG was applicated over 30 min instead of 10 min before ischemia. This longer duration of application resulted in a decrease in left ventricular developed pressure (LVDP) from start until end of the MPG treatment for over 60%. In the present study, no decrease in phasic LVP with 10 min application before ischemia was detectable. In addition, in the current study, a phasic LVP of around 150 mmHg for control and 1 mM-treated animals during preconditioning was measured. In contrast, Tanonaka et al. reported a pre-ischemic LVDP of 75 mmHg in control and 50 mmHg in 1 mM MPG-treated animals [35]. This massive decrease in LVDP by 1 mM MPG may represent an ischemic preconditioning-induced cardioprotection. Taken together, the opposing results to the preconditioning effect of MPG in both studies may arise from different hemodynamic situations due to the study design. We cannot eliminate an impact of the residual antioxidant effects of MPG in the reperfusion phase. However, we would expect that an antioxidative effect of MPG during early phase of reperfusion would be cardioprotective.

mPTP opening triggers cell death and results in a large myocardial infarct size. In the present study, inhibition of mPTP opening with CsA in the presence of Mil and the ROS scavenger MPG reduces infarct sizes compared to Con or Mil and MPG. On the other hand, there is no additive effect of CsA in combination with Mil on infarct size reduction, suggesting that both substances mediate infarct size reduction via the same (inhibition of mPTP opening) and not two different pathways. We cannot conclude an involvement of mPTP opening in Mil-induced protection by our experiments, but we base our assumption on the known involvement of the mPTP in cardioprotection, which is mediated by mBK_Ca_-channels. The activation of mBK_Ca_-channels regulates mitochondrial bioenergetics, leading to an increase in ROS production. This, in turn, serves as a trigger/mediator of the conditioning intervention, resulting in a strong decrease in the detrimental burst of ROS during early reperfusion [31,36]. ROS levels again trigger mPTP opening, which is widely accepted as an endpoint of cardioprotective mitochondrial signaling [7]. In addition, Matsumoto et al. recently showed an involvement of the mPTP in Mil-induced postconditioning. The infarct size reduction by Mil-induced postconditioning was abolished by the mPTP opener atractyloside in an in vivo rat I/R injury model [10]. Taken together, the results of the current study might suggest an inhibition of mPTP opening by Mil preconditioning.

An effect of pentobarbital anesthesia on the preconditioning effect of milrinone can be excluded. All animals received the same anesthesia induction; thus, with respect to infarct size, it must be seen as a systematic error in all groups and could, therefore, not explain the observed differences in infarct size. However, the continuous administration of pentobarbital for anesthesia maintenance has no effect on infarct size reduction by preconditioning interventions [37].

The Langendorff model of an isolated, perfused rat heart was chosen in this study, because of its advantages in studying pathological cardiac conditions such as I/R injury. It is a routinely used model for studying the direct effect of drugs on the heart and making results comparable to prior studies. An alternative approach would have been an in vivo model, but the Langendorff model was preferred for the following reasons. The heart can be investigated without systemic influences from other organs, regulation of circulation or from the nervous system. Therefore, the direct actions of a drug (here milrinone) on the heart (e.g., infarct size) are revealed (reviewed in [38]). On the other hand, the conditions of ischemia and reperfusion, such as duration, are highly reproducible. This is also true for drug application, temperature, buffer conditions and so on. Whether resident immune cell populations contribute to the detected damage or the observed cardioprotection is unclear. There is evidence for an interaction of conditioning interventions/BK_Ca_-channel activation and the immune system. Dai et al. showed that the activation of BK_Ca_-channels reduced I/R-induced leukocyte rolling and adhesion in an intestinal I/R mouse model [39]. However, whether milrinone affects immune cell functions is beyond the scope of the present study.

This study has several limitations. We did not measure ROS levels in our experiments, meaning that we are only able to speculate about differences in ROS levels due to I/R, Mil preconditioning, or MPG application. However, ROS levels increase after reperfusion, while IPC attenuates ROS levels [40]. On the other hand, small amounts of ROS are essential for cardioprotection [41]. Furthermore, we did not verify the involvement of mBK_Ca_-channels in this model, for example, with an mBK_Ca-_channel opener, such as NS1619. Since we already showed that NS1619 preconditioning is cardioprotective in vitro and in vivo, these experiments were deemed unnecessary and therefore unethical [28]. However, a loss of Mil-induced cardioprotection in *KCNMA1^-/-^* mice would give the explicit proof for the involvement of mBK_Ca_-channels in Mil preconditioning. In addition, we omitted a further verification of mPTP involvement, e.g., with application of the mPTP opener atractyloside. For Mil postconditioning, Matsumoto et al. previously demonstrated a loss of infarct size reduction due to atractyloside [10]. Thus, we assumed a similar effect for Mil preconditioning.

Cardioprotective strategies such as preconditioning are of tremendous interest in the hospital setting, in particular to patients suffering from various comorbidities. Many of these comorbidities limit the effectivity of cardioprotective interventions, making it difficult to determine what exactly causes failed cardioprotection. Therefore, this study was designed to determine the underlying mechanisms of milrinone-induced cardioprotection under healthy conditions. In a following step, the exact interactions within the signaling cascade leading to failed cardioprotection in a diseased state should be elucidated.

Taken together, Mil-induced preconditioning reduced infarct size after I/R injury in a concentration-dependent manner and this cardioprotective effect is mediated by the activation of mBK_Ca_-channels, ROS release and inhibition of mPTP opening. Further intermediate steps and possible additional mediators in the signaling cascade of Mil-induced cardioprotection need to be elucidated.

## Figures and Tables

**Table 1:** Weights and ischemic contracture.

	*n*	Body Weight (g)	Heart Weight Dry (g)	Heart Weight Wet (g)	Time of Max. Ischemic Contracture (min)	Level of Max. Ischemic Contracture (mmHg)
Con	9	284 ± 22	0.12 ± 0.02	1.27 ± 0.13	16 ± 2	69 ± 25
Mil 0.3	9	287 ± 14	0.13 ± 0.01	1.37 ± 0.07	16 ± 2	58 ± 12
Mil 1	7	285 ± 20	0.13 ± 0.02	1.33 ± 0.11	17 ± 2	55 ± 12
Mil 3	7	278 ± 18	0.13 ± 0.02	1.36 ± 0.19	18 ± 4	74 ± 22
Mil 10	7	286 ± 28	0.14 ± 0.01	1.33 ± 0.20	17 ± 3	60 ± 18
Con	9	299 ± 17	0.14 ± 0.02	1.35 ± 0.08	17 ± 2	61 ± 9
Mil	9	287 ± 14	0.13 ± 0.01	1.35 ± 0.09	16 ± 2	58 ± 17
Pax + Mil	7	284 ± 19	0.13 ± 0.01	1.33 ± 0.08	17 ± 2	63 ± 14
Pax	9	297 ± 13	0.14 ± 0.02	1.29 ± 0.11	17 ± 1	62 ± 15
MPG + Mil	9	297 ± 14	0.14 ± 0.01	1.35 ± 0.06	16 ± 1	59 ± 15
MPG	9	293 ± 15	0.15 ± 0.02	1.33 ± 0.07	16 ± 1	70 ± 12
MPG + Mil + CsA	9	293 ± 18	0.13 ± 0.01	1.27 ± 0.10	16 ± 2	63 ± 23
Mil + CsA	9	299 ± 19	0.13 ± 0.01	1.32 ± 0.11	16 ± 2	59 ± 18

Data are mean ± SD. Con = Control; Mil = Milrinone; Pax = Paxilline; MPG = N-2-mercaptopropionylglycine; CsA = Cyclosporine A.

**Table 2:** Hemodynamic variables.

	Baseline	PC	Reperfusion	
			30	60
Heart Rate (bpm)				
Con	311 ± 37	302 ± 30	244 ± 46 *	248 ± 34 *
Mil 0.3	313 ± 27	316 ± 32	262 ± 54	285 ± 47
Mil 1	301 ± 21	282 ± 36	252 ± 36	232 ± 68
Mil 3	315 ± 32	344 ± 38	231 ± 78	275 ± 72
Mil 10	325 ± 61	328 ± 45	258 ± 43	263 ± 40
Phasic LVP (mmHg)				
Con	117 ± 20	125 ± 23	26 ± 12 *	27 ± 12 *
Mil 0.3	123 ± 16	123 ± 15	24 ± 11 *	22 ± 7 *
Mil 1	137 ± 9	138 ± 12	17 ± 5 *	29 ± 5 *
Mil 3	129 ± 21	131 ± 19	31 ± 11 *	36 ± 8 *
Mil 10	118 ± 21	130 ± 28	22 ± 14 *	25 ± 12 *
Coronary flow (ml * min^−1^)				
Con	13 ± 3	13 ± 2	7 ± 2 *	6 ± 1 *
Mil 0.3	17 ± 2 ^#^	17 ± 2 ^#^	9 ± 2 *	7 ± 2 *
Mil 1	14 ± 2	14 ± 2	8 ± 1 *	6 ± 1 *
Mil 3	13 ± 4	15 ± 3	8 ± 1 *	7 ± 1 *
Mil 10	14 ± 2	16 ± 2 ^#^	8 ± 1 *	7 ± 1 *

Data are mean ± SD. Con = Control; Mil = Milrinone; PC = Preconditioning; LVP = Left ventricular pressure. * *p* < 0.05 vs. Baseline; ^#^ *p* < 0.05 vs. Con.

**Table 3:** Hemodynamic variables.

	Baseline	PC	Reperfusion	
			30	60
Heart Rate (bpm)				
Con	275 ± 28	264 ± 13	237 ± 65	234 ± 45
Mil	282 ± 34	289 ± 37	260 ± 54	235 ± 46
Pax + Mil	322 ± 29	314 ± 22	280 ± 70	249 ± 40
Pax	298 ± 30	260 ± 36	230 ± 41	233 ± 47
MPG + Mil	301 ± 35	294 ± 39	274 ± 65	227 ± 44
MPG	294 ± 41	284 ± 40	218 ± 71	229 ± 82
MPG + Mil + CsA	314 ± 34	288 ± 42	253 ± 53	222 ± 72
Mil + CsA	299 ± 30	295 ± 26	251 ± 69	204 ± 51
Phasic LVP (mmHg)				
Con	146 ± 19	151 ± 22	18 ± 15 *	21 ± 8 *
Mil	148 ± 23	144 ± 20	23 ± 11 *	32 ± 14 *
Pax + Mil	131 ± 10	122 ± 24 ^#^	25 ± 9 *	35 ± 12 *
Pax	144 ± 24	126 ± 25	30 ± 10 *	35 ± 12 *
MPG + Mil	141 ± 15	157 ± 24	23 ± 15 *	26 ± 16 *
MPG	134 ± 18	148 ± 18	18 ± 7 *	24 ± 8 *
MPG + Mil + CsA	132 ± 33	125 ± 38 ^#^	22 ± 13 *	22 ± 10 *
Mil + CsA	133 ± 22	139 ± 36	26 ± 7 *	33 ± 11 *
Coronary flow (ml * min^−1^)				
Con	16 ± 2	16 ± 3	8 ± 2 *	7 ± 2 *
Mil	16 ± 2	17 ± 2	8 ± 2 *	7 ± 2 *
Pax + Mil	16 ± 4	14 ± 2	9 ± 3 *	8 ± 4 *
Pax	16 ± 2	13 ± 3	8 ± 2 *	7 ± 2 *
MPG + Mil	17 ± 3	19 ± 2	7 ± 2 *	6 ± 2 *
MPG	15 ± 2	17 ± 2	7 ± 3 *	6 ± 3 *
MPG + Mil + CsA	15 ± 3	17 ± 5	6 ± 2 *	5 ± 3 *
Mil + CsA	15 ± 4	16 ± 4	7 ± 2 *	6 ± 2 *

Data are mean±SD. Con = Control; Mil = Milrinone; Pax = Paxilline; MPG = N-2-mercaptopropionylglycine; CsA = Cyclosporine A; PC = Preconditioning; LVP = Left ventricular pressure. * *p* < 0.05 vs. Baseline; ^#^ *p* < 0.05 vs. Con.
